# Cold acclimation of tomato plants is associated with differential expression of genes of the SlLHC family, carotenoid metabolism, and chlorophyll accumulation/degradation

**DOI:** 10.18699/vjgb-26-86

**Published:** 2026-10

**Authors:** M.A. Filyushin, A.V. Shchennikova, E.Z. Kochieva

**Affiliations:** Institute of Bioengineering, Research Center of Biotechnology of the Russian Academy of Sciences, Moscow, Russia; Institute of Bioengineering, Research Center of Biotechnology of the Russian Academy of Sciences, Moscow, Russia; Institute of Bioengineering, Research Center of Biotechnology of the Russian Academy of Sciences, Moscow, Russia

**Keywords:** Solanum lycopersicum L., cold stress, LHC protein family, chlorophyll a/b-binding proteins, carotenoid biosynthesis, ABA, gene expression, Solanum lycopersicum L., холодовой стресс, семейство белков LHC, хлорофилл a/b-связывающие белки, биосинтез каротиноидов, АБК, экспрессия генов

## Abstract

Tomato (Solanum lycopersicum L.) is sensitive to cold stress. The plant’s adaptive response to low temperatures is accompanied by cellular osmoregulation and a decrease in the rate of photosynthesis due to disruption of the antennae of photosystems I and II, including proteins of the light-harvesting complex (LHC) family that bind chlorophylls a/b and carotenoids. In this study, a transcriptome analysis of differential expression of SlLHC family genes, carotenoid and abscisic acid (ABA) biosynthesis genes, as well as ORANGE and STAY-GREEN genes associated with chlorophyll accumulation and degradation, was performed for the first time during two cycles (I and II) of “cold stress (12 h) – recovery (60 h)”. The tomato cultivar Otradnyi was used for the analysis. 12 h before the end of the recovery period of the second cycle (II-48), the content of chlorophylls a/b and carotenoids in plant leaves was shown to be significantly decreased. It was revealed that 12 h of cold exposure in both cycles (I-12, II-12) suppressed the expression of most LHC family genes. At the same time points, a significant increase in the transcript levels of the NSY, CCD-like, LeNCED1 and NCED6 genes associated with ABA synthesis was detected. Genes, expression profile of which differed between the primary and repeated cycles of cold exposure, were identified. Based on these data, a list of candidate genes for cold stress memory markers in plants was compiled: 10 SlLHC family genes (Cab-1A, Cab-1D, CAB12, CAB5-LOC101257186, SEP2, OHP2-LOC101259223, OHP1 and LIL3-2-LOC101247899) and five genes associated with ABA synthesis (VDE, CCD4, CCD-like, LeNCED1 and NCED6). The obtained results are important for identifying and understanding the involvement of photosynthesis-related genes in the response to cold stress and determining cold tolerance of tomato plants, and can be used in breeding cold-resistant varieties.

## Abstract (ru)

Растения томата (Solanum lycopersicum L.) чувствительны к воздействию холодового стресса, адаптационный ответ на который связан с клеточной осморегуляцией и снижением скорости фотосинтеза за счет нарушения работы антенн фотосистем I и II, включающих белки семейства Light-Harvesting Complex (LHC), связывающие хлорофиллы a/b и каротиноиды. В настоящей работе впервые проведен транскриптомный анализ дифференциальной экспрессии генов семейства SlLHC, генов биосинтеза каротиноидов и абсцизовой кислоты (АБК), а также генов ORANGE и STAY-GREEN, связанных с синтезом и деградацией хлорофиллов, в динамике двух циклов (I и II) «холодовой стресс (12 ч) – восстановление (60 ч)». Для анализа использован сорт томата Отрадный. Показано, что за 12 ч до завершения периода восстановления второго цикла (II-48) в листьях растения значительно уменьшается количество хлорофиллов a/b и каротиноидов. Выявлено, что 12 ч холодового воздействия в обоих циклах (I-12, II-12) подавляют экспрессию большинства генов семейства LHC. В тех же точках обнаружено значимое повышение уровня транскриптов генов NSY, CCD-like, LeNCED1 и NCED6, связанных с синтезом АБК. Определены гены, профиль экспрессии которых различается между первичным и повторным циклами холодового воздействия. На основании этих данных составлен список генов-кандидатов в маркеры памяти растений о холодовом стрессе: десять генов семейства SlLHC (Cab-1A, Cab-1D, CAB12, CAB5-LOC101257186, SEP2, OHP2-LOC101259223, OHP1 и LIL3-2-LOC101247899) и пять генов, ассоциированных с синтезом АБК (VDE, CCD4, CCD-like, LeNCED1 и NCED6). Полученные результаты важны для определения и осмысления участия генов, связанных с фотосинтезом, в ответе на холодовой стресс и определении холодостойкости растений томата, и могут быть использованы в селекции холодостойких сортов.

## Introduction

The survival, productivity, and geographic distribution of plants are limited by unfavorable environmental conditions. Among the most serious factors, cold stress can have both less destructive (chilling) and more destructive (freezing) forms (Kidokoro et al., 2022). Unable to move, plants are forced to adapt to stressful conditions, including low temperatures (Browse, Xin, 2001), which is accompanied by physiological and molecular genetic changes (Ding et al., 2024). Primary stress triggers epigenetic modifications in the genome, thus improving plant adaptation to recurrent events and forming stress memory (Villagómez-Aranda et al., 2022). Genes that exhibit differences in expression patterns between priming and repeats, as well as during recovery periods, are considered markers of stress memory (Friedrich et al., 2019; Bäurle, Trindade, 2020; Jacques et al., 2021; Aina et al., 2024).

In the plant response to cold stress, one of the significant groups of genes is associated with photosynthesis, the reduction of which stimulates cellular osmoregulation (Yamori et al. 2014). The latter is achieved by stomatal closure under the action of abscisic acid (ABA) (Sreenivasulu et al., 2012; Agurla et al., 2018), formed during the oxidative cleavage of the carotenoid 9′-cis-neoxanthin (Nakashima, Yamaguchi- Shinozaki, 2013; Chen et al., 2020; Xu et al., 2023).

Cold-induced inhibition of photosynthesis is characterized by impaired light energy capture by the light-harvesting complexes (antennae) of photosystems I and II and electron transfer in thylakoids (Ashraf, Harris, 2013). The antennae include proteins of the Light-Harvesting Complex (LHC) family, which are embedded in the thylakoid membrane and noncovalently bind to chlorophylls a and/or b, altering their optical properties (Klimmek et al., 2006). The resulting set of different chlorophyll forms ensures the transfer of energy from absorbed quanta to the reaction centers of the photosystems (Li et al., 2025). Also, LHC proteins are involved in chlorophyll degradation by interacting with the STAY-GREEN (SGR) protein and chlorophyll catabolism enzymes (CCE) (Pietrzykowska et al., 2014). The LHC family includes chlorophyll a/b-binding proteins (CAB or Lhca/b), photosystem II protein psbS, Light- Harvesting-like proteins (LIL, also including Light-Inducible proteins (ELIP), One-Helix proteins (OHP), Stress-Enhanced proteins (SEP)), and ferrochelatases (FC or FeCh) (Jansson, 1999; Klimmek et al., 2006).

In addition to LHC proteins and chlorophylls a/b, antenna complexes include carotenoids (β-carotene, xanthophylls of the lutein and violaxanthin cycles), which are involved in the capture of light quanta in spectral ranges weakly absorbed by chlorophylls, followed by the transfer of excitation energy to chlorophyll molecules (Li et al., 2025). Furthermore, carotenoids (primarily violaxanthin, antheroxanthin, and zeaxanthin) protect the photosynthetic apparatus from excess excitation energy and prevent its destruction by neutralizing singlet oxygen and triplet chlorophyll (Li et al., 2025).

The ORANGE (OR) protein plays an important role in regulating chlorophyll and carotenoid levels in green plant tissues. For example, the expression level of SlOR and SlOR-like genes in the stems and leaves of tomato Solanum lycopersicum L. is positively associated with the levels of chlorophylls and carotenoids in leaf cells (Gao et al., 2025). However, overexpression of the OR gene in the model species Arabidopsis thaliana L. suppresses chlorophyll biosynthesis and thylakoid membrane development (Tee, 2019).

The LHC gene family has been identified in various plant species, including A. thaliana (30, 32, or 34 genes), poplar Populus spp. (36 or 39), cotton Gossypium hirsutum L. (86), and apple Malus × domestica Borkh. (27 or 42) (Klimmek et al., 2006; Zhao et al., 2020; Zhang et al., 2021; Lan et al., 2022).

Tomato plants (S. lycopersicum), an important vegetable crop, are sensitive to temperatures below 12 °C (Mesa et al., 2022) and are therefore grown primarily indoors in most regions of the Russian Federation due to the risk of sudden temperature fluctuations and recurrent spring cold spells or frosts. During the development of stress resistance, the parameters of photosynthesis undergo significant changes, including the expression of photosynthetic proteins (Neilson et al., 2011; Soualiou et al., 2023), so studying tomato LHC genes may be promising for the breeding of new varieties.

The number of LHC genes in S. lycopersicum, as in other plant species, is not precisely defined and depends on the specific study: 40 (Lan et al., 2022) or 39 (Meng et al., 2025). Most LHC genes contain stress-associated cis-regulatory elements in their promoters. These genes are differentially expressed in response to drought, cold, and salinity, may play an important role in many biological processes, and are shown to be regulated by circadian rhythms (Piechulla et al., 1998; Lan et al., 2022; Meng et al., 2025). The transcript level of SlLHC genes increases during plant adaptation to salt stress (Ikuyinminu et al., 2023) and is positively related to the rate of photosynthesis (Yan et al., 2023).

The aim of this study was a transcriptome analysis of leaves of the cold-resistant tomato cultivar Otradnyi in terms of changes in the expression of LHC genes, genes of the carotenoid biosynthesis pathway, as well as genes associated with the synthesis of carotenoids and chlorophyll in chloroplasts, the formation of ABA and the degradation of chlorophylls, in response to two cycles of “cold stress – post-stress recovery”. The results obtained are important for understanding the involvement of photosynthesis-related genes in the cold stress response and cold tolerance of tomato plants, with the potential to identify markers of cold stress memory among these genes.

## Materials and methods

In the study, we used plants of the tomato S. lycopersicum cold-resistant cv. Otradnyi (code 8006741, https://gossortrf. ru/registry/) bred by the Federal Scientific Vegetable Center (FSVC, Moscow Region). This cultivar is early ripening and resistant to salinity (Filyushin et al., 2025; data from the originators).

Tomato cv. Otradnyi seeds (the 2024 harvest) were used to obtain seedlings at the 5–7 leaf stage (light/dark – 16 h/8 h, 23 °C/18 °C; dark phase 23:00–7:00; Photosynthetic Photon Flux (PPF) – 190 μM/(m2 ‧ s)). Plants were subjected to two cycles of cold stress; controls were incubated under normal conditions (23 °C). Each cycle consisted of a cooling phase of 12 h at 3 °C (20:00–8:00) (I-12 and II-12) followed by a poststress recovery period (memory phase) of 60 h under normal conditions (Fig. 1). The cycles followed one after another and in total took 144 h (12+60+12+60).

**Fig. 1.**
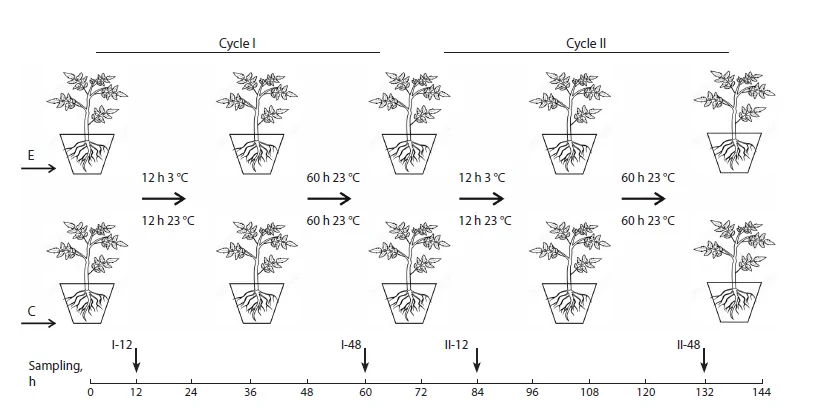
Schematic representation of two-cycle (I, II) exposure to cold stress with subsequent recovery on tomato plants of the cv. Otradnyi.

The use of two cycles was motivated by the signature of stress memory marker genes, implying differences in the expression patterns of such genes between priming and re-exposure (Friedrich et al., 2019; Bäurle, Trindade, 2020; Jacques et al., 2021; Aina et al., 2024). That is, if the expression profile of the genes we analyzed under two-cycle stress shows significant intercycle differences, such genes can become candidates for stress memory markers. In addition, we used a 60-h recovery period, since another characteristic of stress memory marker genes is the failure to return to normal expression values for a long time (more than 24 h) after stress (Friedrich et al., 2019; Bäurle, Trindade, 2020; Jacques et al., 2021; Aina et al., 2024).

Over the course of two cycles (a total of 144 h), no visible differences were found between the experimental plants and the controls. In both cold treatment cycles, no signs of leaf wilting were observed in the stressed samples (apparently due to the cold tolerance of the cv. Otradnyi). There were also no significant differences in plant height, leaf number and size between the control and experimental groups, as well as within them.

At the end of cold exposure (12 h, 8:00) and after 48 h of recovery (8:00) of the first (I) and second (II) cycles, leaf samples were collected from the experimental and control plants. In the recovery phase (60 h), a 48-h point (I-48 and II-48) was chosen, 12 h before the end of the post-stress period, so that sampling was carried out at the same time of day (8:00) both during stress and recovery. The choice was determined by the dependence of the expression of the analyzed photosynthesis genes on circadian rhythms (Piechulla et al., 1998; Meng et al., 2025). This temporal coordination, combined with a parallel cultured control for normalization, should improve the accuracy of interpretation of the resulting biochemical and transcriptomic data.

The samples were analyzed for chlorophylls a/b and carotenoids (two biological and three analytical replicates); one biological replicate was a pool of leaves from two independent plants for each measurement point. The concentrations (mg/g fresh weight (FW)) of chlorophylls a and b and total carotenoids were determined in chloroform-methanol (3:1, v/v) extracts according to (Efremov et al., 2020), with absorption spectra recorded on an Eppendorf BioSpectrometer® basic (Eppendorf, Germany). The following formulas were used to calculate pigment concentrations:

**Formula. 1.**
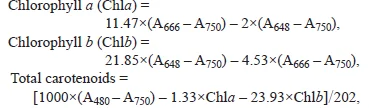
Formula 1

where A666, A648, A750 and A480 are the absorption values at the indicated wavelengths

The significance (p < 0.05) of differences between values was determined using Two-way ANOVA (“multiple comparisons, corrected with Bonferroni test”) in GraphPad Prism v. 8 (“GraphPad Software Inc.”, USA; https://www.graphpad.com/ scientific-software/prism/).

For subsequent transcriptome analysis, total RNA was isolated from the same leaf samples (RNeasy Plant Mini Kit, Qiagen, USA) and used to obtain mRNA libraries (NEBNext® mRNA Library Prep Reagent Set for Illumina; New England BioLabs, USA), which were sequenced on the Illumina HiSeq2500 platform (Illumina Inc., USA). Based on the obtained reads, coding sequences were assembled using Trinity v3.5.13 (https://github.com/trinityrnaseq/trinityrnaseq/wiki) and TransDecoder v5.1.0 (https://github.com/TransDecoder/ TransDecoder) and then annotated with NCBI-Blast (https:// www.ncbi.nlm.nih.gov/). Relative transcript levels were estimated as RPKM (number of reads per 1 kb transcripts per million mapped reads).

Differentially expressed genes (DEGs) between the control and experimental plants in two cycles at the end of stress (I- 12, II-12) and the recovery period (I-48, II-48) were identified using the DESeq2 method (Love et al., 2014). Differences in transcript levels were considered significant if the expression change was ≥2 (absolute log2FoldChange [FC] ≥ 1; padj (P-value adjusted, or false discovery rate) <0.05). Next, the annotation was used to search for tomato LHC superfamily genes, carotenoid metabolism pathway genes, as well as OR, OR-like, and SGR genes, both among the primary gene list and among the DEGs. Changes in gene expression profiles during the experiment were visualized using heatmaps (Excel).

## Results

In this study, we examined the effect of two “cold stress – recovery” cycles (I, II) on metabolite content and photosynthesisrelated gene expression in leaves of the cold-tolerant tomato cv. Otradnyi. Leaves were collected from experimental plants (parallel to control samples) at two points in each cycle: at the end of the 12-h stress period (I-12, II-12); and 12 h before the end of the 60-hour recovery phase (I-48, II-48).

Tissue samples were used for chlorophyll a and b and total carotenoid content analysis. At point I-48, both in the control and the experimental group, the obtained values were significantly lower than at the other three points (II-48, I-12, and II-12). The exception was carotenoids: their content at point I-48 differed from I-12, but not from II-12, but was similar in the control and experimental groups (Fig. 2). In addition, in the experimental group, the amount of all analyzed pigments decreased significantly at the end of the recovery period of the second cycle (II-48) (Fig. 2).

**Fig. 2.**
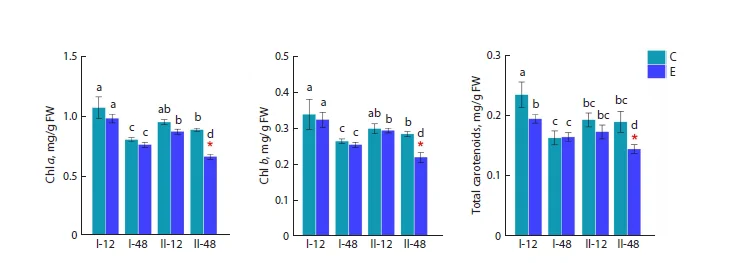
Dynamics of the content (mg/g FW) of chlorophylls a and b and total carotenoids in the leaves of tomato cv. Otradnyi plants in response to cold stress and recovery. In each of the two exposure cycles (I, II), measurements were taken at the end of stress exposure (I-12, II-12) and after 48 h of the recovery period (I-48, II-48). The value a–d p < 0.05 – for statistically significant differences between the experiment (E) and the control (C) at each of the points; a significant difference between the experiment and the control at point II-48 is additionally indicated by a red asterisk.

The same leaf samples were used to obtain transcriptomes for subsequent analysis of the expression dynamics of photosynthesis- related genes. A preliminary list of genes of interest was compiled based on transcriptome annotations from SolGenomics, including studies (Lan et al., 2022; Meng et al., 2025), and NCBI (see the Table). The former study identified 24 Lhca/b, 10 Lil, one Psbs, and five FCII genes in tomato (Lan et al., 2022). The latter publication reported 29 Lhca/b, seven Lil, one Psbs, and two FCII genes (Meng et al., 2025). We further relied on NCBI data, where the current version of the tomato genome is dated September 3, 2024, compared to SolGenomics (September 2019).

**Table 1.**
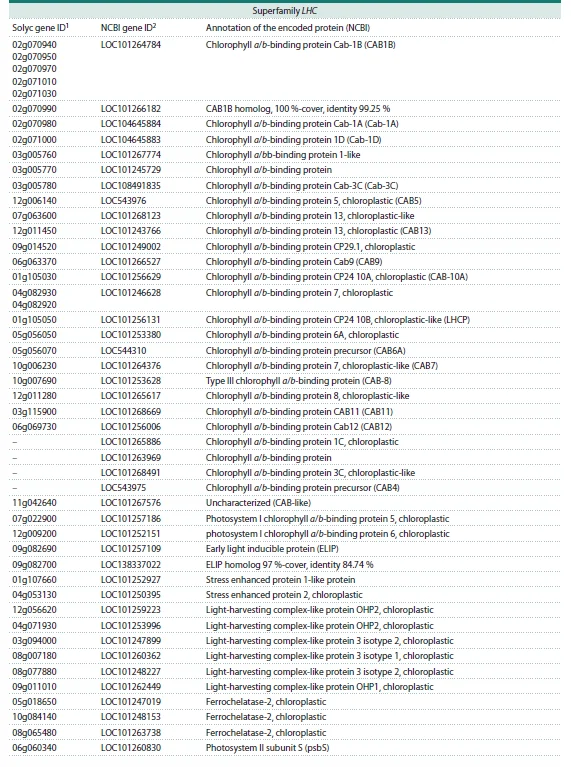
List of genes analyzed in the study

**Tab1end.**
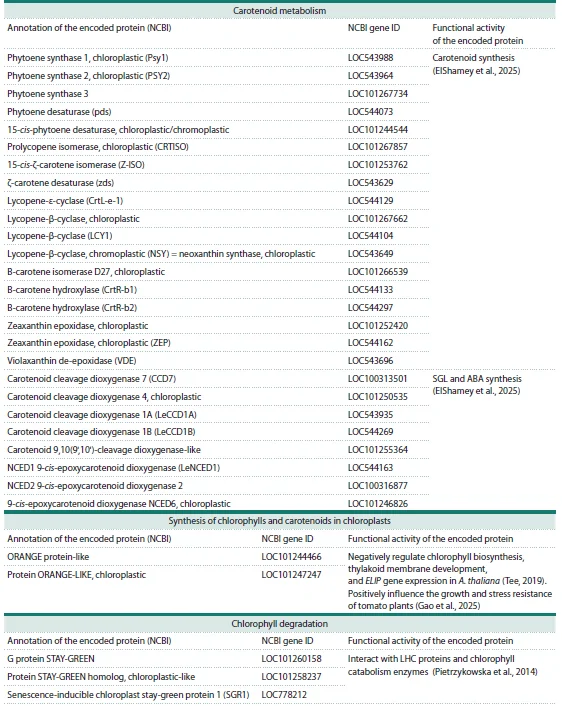
Table 1end 1 Current Tomato Genome version SL4.0 and Annotation ITAG4.0, from September 2019 (https://www.solgenomics.net/organism/Solanum_lycopersicum/ genome), using data of (Lan et al., 2022; Meng et al., 2025). 2 SLM_r2.1 (GCF_036512215.1), assembly and annotation of the tomato genome, September 3, 2024 (updates October 2024–September 2025; https://www.ncbi.nlm.nih.gov/datasets/genome/GCF_036512215.1/).

According to NCBI data, the tomato LHC family includes 43 genes (29 – Lhca/b, 10 – Lil, 1 – psbS, 3 – FCII) (see the Table). In addition, 18 genes of the carotenoid biosynthesis pathway, eight deoxygenase genes (catalyzing the conversion of carotenoids to the phytohormones strigolactones (SGL) and ABA), two homologous OR genes (synthesis of chlorophylls and carotenoids in chloroplasts) and three homologous SGR genes (chlorophyll degradation) were identified in tomato genome (see the Table).

The obtained transcriptome data were used to assess changes in the expression of the genes we selected (see the Table) in tomato leaves during two cycles of “cold stress – recovery” (Fig. 3 and 4).

**Fig. 3.**
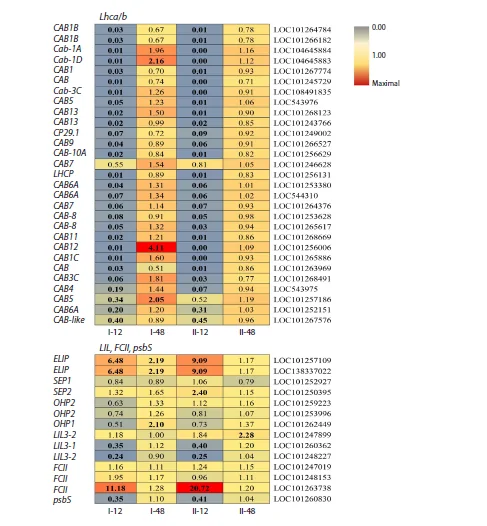
Heatmap of changes in LHC superfamily gene expression in the leaves of the tomato cv. Otradnyi (S. lycopersicum) in response to two cycles (I, II) of “cold stress – recovery”, constructed using our transcriptome data for points I-12, II-12 (end of stress) and I-48, II-48 (recovery period). Here and in Figure 4: cells show RPKM values rounded to two decimal places; statistically significant differences from the control (taken as 1.00) are highlighted in bold. The cell color gradient corresponds to a decrease (shades of gray) or increase (shades of red) in gene expression compared to the control (1.00, yellow color).

**Fig. 4.**
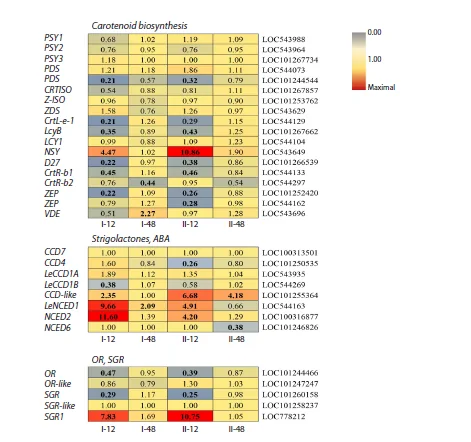
Heatmap of changes in the expression of carotenoid, SGL and ABA biosynthesis genes, as well as OR and SGR genes, in the leaves of tomato cv. Otradnyi in response to two cycles (I, II) of “cold stress – recovery”. Constructed using the transcriptome data obtained for points I-12, II-12 (end of stress) and I-48, II-48 (recovery period).

It was shown that in both stress cycles after 12 h of cold exposure (I-12, II-12), the expression of Lhca/b genes decreased significantly (≥2-fold). The exceptions were CAB7- LOC101246628 (I-12, II-12) and CAB5-LOC101257186 (II-12), the level of transcripts of which also decreased at both points, however, less significantly (<2-fold). By the end of the recovery period of both cycles (I-48, II-48), the expression of Lhca/b genes returned to control values, with the exception of an increased level of Cab-1D, CAB12 and CAB5-LOC101257186 transcripts (I-48) (Fig. 3).

Among the other gene groups of the LHC family, the expression of both the ELIP genes and one of the ferrochelatases (LOC101263738) increases significantly towards the end of stress (I-12, II-12) and after recovery maintains an elevated value (I-48) or returns to the control level (II-48). The expression of two LIL3 genes (LOC101260362, LOC101248227) and the psbS and OHP1 genes, on the contrary, decreases in response to stress (I-12, II-12) and, upon recovery, returns to the control (I-48, II-48; except for the induction of OHP1 in I-48). The remaining genes showed no significant changes in expression across the two stress – recovery cycles, with the exception of SEP2 (upregulation, II-12) and one of LIL3-2- LOC101247899 (upregulation, II-48) (Fig. 3).

The previously formulated stress memory marker feature states that markers are genes, expression of which changes differently in response to the first and repeated stressful events, and to the recovery periods separating them (Friedrich et al., 2019; Bäurle, Trindade, 2020; Jacques et al., 2021). Given this, the Cab-1A, Cab-1D, CAB12, CAB5-LOC101257186, ELIP, SEP2, OHP2-LOC101259223, OHP1 and LIL3-2- LOC101247899 genes, which showed intercycle differences in expression dynamics, may be considered candidates for such markers (Fig. 3).

In addition to the LHC family genes, transcriptome analysis of the carotenoid biosynthesis genes was performed. As a result, no significant response to both “cold stress – recovery” cycles was found for the genes of all three phytoene synthases (PSY1–3), one of the two PDS-LOC544073, CRTISO, Z-ISO, ZDS, and LCY1 (Fig. 4). The second PDS-LOC101244544, CrtL-e-1, LcyB, D27, CrtR-b1, and one of the two ZEP genes showed the same expression dynamics: a decrease (I-12, II-12) and a return to the control level (I-48, II-48); the expression of the second ZEP gene had similar dynamics, except for a less significant decrease at point I-12 (Fig. 4). The level of CrtR- b2 transcripts, on the contrary, decreased after the recovery periods (I-48, II-48). Two genes deserve special mention. The expression of the first one encoding neoxanthin synthase (NSY ) was significantly increased in response to cold exposure (I-12, II-12) and returned to control levels after the recovery period (I-48, II-48). The level of transcripts of the second gene encoding violaxanthin de-epoxidase (VDE) decreased in the first stress cycle (I-12), and then increased (I-48), and in the second cycle did not differ from the control (Fig. 4).

Among the genes encoding dioxygenases involved in the pathway for converting carotenoids into phytohormones (SGL, ABA), CCD7 and LeCCD1A showed no significant response to two “cold stress – recovery” cycles. CCD4 and NCED6 expression differed from the control profile only by a decrease at points II-12 and II-48, respectively. The transcript levels of the LeCCD1B and NCED2 genes significantly decreased and increased, respectively, after stress (I-12, II-12). The CCD-like and LeNCED1 genes had transcript levels significantly higher than the control, except at points I-48 and II-48, respectively (Fig. 4).

Of the genes associated with chlorophyll synthesis and degradation, two (OR-like, SGR-like) showed no differences from the control, two (OR, SGR) had significantly reduced transcript levels after stress (I-12, II-12), and one (SGR1) had an increased level at the same points (I-12, II-12) (Fig. 4).

Given the definition of a stress memory marker (Friedrich et al., 2019; Bäurle, Trindade, 2020; Jacques et al., 2021), the following genes may be considered candidates: VDE, CCD4, CCD-like, LeNCED1, and NCED6 (Fig. 4).

## Discussion

The plant adaptive response to cold stress is closely linked to cellular osmoregulation mediated by ABA-induced stomatal closure (Sreenivasulu et al., 2012; Agurla et al., 2018). 9′-cisneoxanthin serves as a precursor of ABA, and its increased synthesis in response to stress can be accompanied by a decrease in the content of violaxanthin cycle xanthophylls important for photosynthesis, leading to a decrease in the rate of photosynthesis (Nakashima, Yamaguchi-Shinozaki, 2013; Yamori et al. 2014; Chen et al., 2020; Xu et al., 2023). This disrupts the function of the antennae of photosystems I and II (Ashraf, Harris, 2013), which include proteins of the LHC family that bind chlorophylls a/b and carotenoids (Klimmek et al., 2006; Li et al., 2025). Studying LHC genes is therefore important for understanding their role in the cold stress response and determining plant cold tolerance.

Since the available information on the S. lycopersicum LHC genes varies (Lan et al., 2022; Meng et al., 2025), the first step was the identification of genes of this family using the above-mentioned publications and GenBank data (SolGenomics and NCBI). The results obtained differed from those of the studies (Lan et al., 2022; Meng et al., 2025) that used only the SolGenomics database, both in terms of the number and composition of the LHC family (see the Table). Our data are believed to be the most reliable since they are based on the latest version of the S. lycopersicum genome available in the NCBI database (from 2024 vs. 2019 in SolGenomics).

Considering the involvement of carotenoids in photosynthesis, the list of genes for analysis also included known genes of the carotenoid metabolism pathway (ElShamey et al., 2025), as well as SGR and OR genes associated with chlorophyll degradation (Pietrzykowska et al., 2014) and chlorophyll/carotenoid synthesis (Tee, 2019; Gao et al., 2025), respectively (see the Table).Having selected the cold-resistant cv. Otradnyi for the study, we carried out two consecutive “cold stress – recovery” cycles (I, II), where each cycle included 12-h cooling (3 °C) followed by 60 h of normal conditions (Fig. 1). Since the level of LHC gene transcripts is regulated by circadian rhythms (Piechulla et al., 1998; Meng et al., 2025), we used control plants grown in parallel at normal temperature (23 °C), taking readings at the same points as the experiment.

Measurement of chlorophyll a/b and carotenoid contents in leaves collected at the end of the 12-h stress period (I-12, II-12) and during the recovery phase (I-48, II-48) showed that the amounts of all analyzed pigments decreased significantly at the end of the second cycle (II-48) (Fig. 2). This may suggest a more effective plant response to repeated cold stress due to a possible decrease in the rate of photosynthesis and an increase in osmoregulation, in accordance with data (Sreenivasulu et al., 2012; Agurla et al., 2018). That is, stress memory may have occurred in plants during the first cycle “cold stress – recovery”, which could be reflected in transcriptomic data.

The same tomato leaf samples were used to obtain transcriptomes and analyze changes in the expression of genes of interest (see the Table): LHC family, carotenoid metabolism, SGR and OR genes. The observed decrease in transcript levels of most LHC genes and half of the carotenoid biosynthesis genes at the end of the stress exposure (I-12, II-12) (Fig. 3 and 4) may suggest a decrease in the rate of photosynthesis in tomato leaves in response to low temperature, since the rate of photosynthesis is positively associated with LHC gene expression (Yan et al., 2023). A sharp increase in the expression of the SGR1 gene (Fig. 4), involved in chlorophyll degradation (Pietrzykowska et al., 2014), and a decrease in the expression of the OR gene (Fig. 4), positively associated with chlorophyll biogenesis and the development of thylakoid membranes (Tee, 2019), may also be associated with a decrease in photosynthesis. At the same time, a significant increase in the expression of the NSY, CCD-like, LeNCED1, and NCED6 genes (Fig. 4) in I-12 and II-12 leaves may suggest increased ABA production (ElShamey et al., 2025), associated with stomatal closure in response to cold stress (Sreenivasulu et al., 2012; Agurla et al., 2018).

Previously, it was shown that the level of S. lycopersicum LHC gene transcripts increases during tomato plant adaptation to salt stress (Ikuyinminu et al., 2023). The discrepancy with our data (Fig. 3) can be explained both by the use of a different stress factor and by the use of the biostimulant PSI-475 by the authors before watering the plants with a saline solution (Ikuyinminu et al., 2023).

The publication (Meng et al., 2025) also presents data on the expression of S. lycopersicum LHC genes in response to a single-cycle 24-h cold stress. In particular, it was shown that the level of SlLhcb1.11 (LOC108491835) gene transcripts decreases and SlELIP1 (LOC101257109) increases (Meng et al., 2025), which is completely consistent with our results (Fig. 3). Silencing of SlLhcb1.11 or SlELIP1 in tomato plants leads to a decrease in chlorophyll content, impaired photosynthesis and cold resistance (Meng et al., 2025), indicating an important role of these genes in the adaptive response of S. lycopersicum. According to our data, the SlELIP1 gene may be a candidate for stress memory markers, since its expression profile differs between the first and second recovery periods (Fig. 3). In addition to SlELIP1, a number of genes correspond to the stress memory marker definition (Friedrich et al., 2019; Bäurle, Trindade, 2020; Jacques et al., 2021): Cab-1A, Cab-1D, CAB12, CAB5-LOC101257186, SEP2, OHP2-LOC101259223, OHP1 and LIL3-2-LOC101247899, VDE, CCD4, CCD-like, LeNCED1 and NCED6 (Fig. 3 and 4).

## Conclusion

Overall, in this study, we performed the first transcriptome analysis of the differential expression of SlLHC genes, carotenoid metabolism genes, and genes associated with chlorophyll synthesis and degradation in the leaves of a coldtolerant tomato genotype during two consecutive cycles “cold stress – recovery”. Signs of a possible decrease in the rate of photosynthesis were obtained: a decrease in the chlorophyll and carotenoid content at the end of the second cycle recovery (II-48) and a decrease in the transcript levels of most LHC family genes at the end of each chilling stage (I-12, II-12). At points I-12 and II-12, an increase in the expression of the NSY, CCD-like, LeNCED1, and NCED6 genes was also detected, which may indicate a possible increase in ABA synthesis in response to low temperature. Based on the obtained expression dynamics, among the analyzed genes, candidates for the role of markers of tomato cold stress memory were identified, namely 10 SlLHC genes and five genes associated with ABA synthesis.

## Conflict of interest

The authors declare no conflict of interest.

## References

Agurla S., Gahir S., Munemasa S., Murata Y., Raghavendra A.S.
Mechanism
of stomatal closure in plants exposed to drought and cold
stress. In: Iwaya-Inoue M., Sakurai M., Uemura M. (Eds) Survival
Strategies in Extreme Cold and Desiccation. Advances in Experimental
Medicine and Biology. Vol. 1081. Springer, 2018;215-232.
doi 10.1007/978-981-13-1244-1_12

Aina O., Bakare O.O., Fadaka A.O., Keyster M., Klein A. Plant biomarkers
as early detection tools in stress management in food crops:
a review. Planta. 2024;259(3):60. doi 10.1007/s00425-024-04333-1

Ashraf M., Harris P.J.C. Photosynthesis under stressful environments:
an overview. Photosynthetica. 2013;51:163-190. doi 10.1007/
s11099-013-0021-6

Bäurle I., Trindade I. Chromatin regulation of somatic abiotic stress
memory. J Exp Bot. 2020;71(17):5269-5279. doi 10.1093/jxb/
eraa098

Browse J., Xin Z. Temperature sensing and cold acclimation. Curr Opin
Plant Biol. 2001;4:241-246. doi 10.1016/s1369-5266(00)00167-9

Chen K., Li G.J., Bressan R.A., Song C.P., Zhu J.K., Zhao Y. Abscisic
acid dynamics, signaling, and functions in plants. J Integr Plant Biol.
2020;62(1):25-54. doi 10.1111/jipb.12899

Ding Y., Shi Y., Yang S. Regulatory networks underlying plant responses
and adaptation to cold stress. Annu Rev Genet. 2024;58(1):43-65.
doi 10.1146/annurev-genet-111523-102226

Efremov G.I., Slugina M.A., Shchennikova A.V., Kochieva E.Z. Differential
regulation of phytoene synthase PSY1 during fruit carotenogenesis
in cultivated and wild tomato species (Solanum section
Lycopersicon). Plants. 2020;9(9):1169. doi 10.3390/plants9091169

ElShamey E., Zeng Y., Ding Y., Yang J. Functional phytochemicals in
tomatoes: biosynthesis, gene regulation, and human health implications.
Front Plant Sci. 2025;16:1662388. doi 10.3389/fpls.2025.
1662388

Filyushin M.A., Shchennikova A.V., Kochieva E.Z. Differential expression
profile of DREB2 subfamily transcription factor genes in the
dynamics of salt stress and post-stress recovery in tomato plants.
Vavilovskii Zhurnal Genetiki i Selektsii = Vavilov J Genet Breed.
2025;29(8):1213-1220. doi 10.18699/vjgb-25-128

Friedrich T., Faivre L., Bäurle I., Schubert D. Chromatin-based mechanisms
of temperature memory in plants. Plant Cell Environ. 2019;
42(3):762-770. doi 10.1111/pce.13373

Gao Y., Zhou X., Huang H., Wang C., Xiao X., Wen J., Wu J., Zhou S.,
de Dios V.R., Rodríguez L.G., Yao Y., Liu J., Deng H. ORANGE
proteins mediate adaptation to high light and resistance to Pseudomonas
syringae in tomato by regulating chlorophylls and carotenoids
accumulation. Int J Biol Macromol. 2025;306(Pt.4):141739. doi
10.1016/j.ijbiomac.2025.141739

Ikuyinminu E., Goñi O., Łangowski Ł., O’Connell S. Transcriptome,
biochemical and phenotypic analysis of the effects of a precision
engineered biostimulant for inducing salinity stress tolerance in tomato.
Int J Mol Sci. 2023;24(8):6988. doi 10.3390/ijms24086988

Jacques C., Salon C., Barnard R.L., Vernoud V., Prudent M. Drought
stress memory at the plant cycle level: a review. Plants (Basel).
2021;10(9):1873. doi 10.3390/plants10091873

Jansson S. A guide to the Lhc genes and their relatives in Arabidopsis.
Trends Plant Sci. 1999;4(6):236-240. doi 10.1016/s1360-1385(99)
01419-3

Kidokoro S., Shinozaki K., Yamaguchi-Shinozaki K. Transcriptional
regulatory network of plant cold-stress responses. Trends Plant Sci.
2022;27(9):922-935. doi 10.1016/j.tplants.2022.01.008

Klimmek F., Sjödin A., Noutsos C., Leister D., Jansson S. Abundantly
and rarely expressed Lhc protein genes exhibit distinct regulation
patterns in plants. Plant Physiol. 2006;140(3):793-804. doi 10.1104/
pp.105.073304

Lan Y., Song Y., Zhao F., Cao Y., Luo D., Qiao D., Cao Y., Xu H.
Phylogenetic, structural and functional evolution of the LHC gene
family in plant species. Int J Mol Sci. 2022;24(1):488. doi 10.3390/
ijms24010488

Li Y., Cao T., Guo Y., Grimm B., Li X., Duanmu D., Lin R. Regulatory
and retrograde signaling networks in the chlorophyll biosynthetic
pathway. J Integr Plant Biol. 2025;67(4):887-911. doi 10.1111/
jipb.13837

Love M.I., Huber W., Anders S. Moderated estimation of fold change
and dispersion for RNA-seq data with DESeq2. Genome Biol. 2014;
15(12):550. doi 10.1186/s13059-014-0550-8

Meng F., Ma M., Li S., Liang P., Liang Y., Shi H., Huang S., Su H.,
Deng Y., Akram M.A., Shen X., Feng R., Zhan X., Ma F. Genomewide
identification of light-harvesting chlorophyll a/b-binding
(LHC) gene family in tomato and functional analysis of SlLhcb1.11
and SlELIP1 under cold stress. Genomics. 2025;117(2):111022. doi
10.1016/j.ygeno.2025.111022

Mesa T., Polo J., Arabia A., Caselles V., Munné-Bosch S. Differential
physiological response to heat and cold stress of tomato plants and its
implication on fruit quality. J Plant Physiol. 2022;268:153581. doi
10.1016/j.jplph.2021.153581

Nakashima K., Yamaguchi-Shinozaki K. ABA signaling in stress-response
and seed development. Plant Cell Rep. 2013;32(7):959-970.
doi 10.1007/s00299-013-1418-1

Neilson K.A., Mariani M., Haynes P.A. Quantitative proteomic analysis
of cold-responsive proteins in rice. Proteomics. 2011;11(9):1696-
1706. doi 10.1002/pmic.201000727

Piechulla B., Merforth N., Rudolph B. Identification of tomato Lhc promoter
regions necessary for circadian expression. Plant Mol Biol.
1998;38(4):655-662. doi 10.1023/a:1006094015513

Pietrzykowska M., Suorsa M., Semchonok D.A., Tikkanen M., Boekema
E.J., Aro E.-M., Jansson S. The light-harvesting chlorophyll a/b
binding proteins Lhcb1 and Lhcb2 play complementary roles during
state transitions in Arabidopsis. Plant Cell. 2014;26(9):3646-3660.
doi 10.1105/tpc.114.127373

Soualiou S., Duan F., Li X., Zhou W. Nitrogen supply alleviates cold
stress by increasing photosynthesis and nitrogen assimilation in
maize seedlings. J Exp Bot. 2023;74(10):3142-3162. doi 10.1093/
jxb/erad073

Sreenivasulu N., Harshavardhan V.T., Govind G., Seiler C., Kohli A.
Contrapuntal role of ABA: does it mediate stress tolerance or plant
growth retardation under long-term drought stress? Gene. 2012;
506(2):265-273. doi 10.1016/j.gene.2012.06.076

Tee E.E. Orange is the new green: Arabidopsis ORANGE represses
chloroplast biogenesis. Plant Cell. 2019;31(12):2824-2825. doi
10.1105/tpc.19.00670

Villagómez-Aranda A.L., Feregrino-Pérez A.A., García-Ortega L.F.,
González-Chavira M.M., Torres-Pacheco I., Guevara-González R.G.
Activating stress memory: eustressors as potential tools for plant
breeding. Plant Cell Rep. 2022;41(7):1481-1498. doi 10.1007/
s00299-022-02858-x

Xu C., Wang Y., Yang H., Tang Y., Liu B., Hu X., Hu Z. Cold acclimation
alleviates photosynthetic inhibition and oxidative damage
induced by cold stress in citrus seedlings. Plant Signal Behav. 2023;
18(1):2285169. doi 10.1080/15592324.2023.2285169

Yamori W., Hikosaka K., Way D.A. Temperature response of photosynthesis
in C3, C4, and CAM plants: temperature acclimation and
temperature adaptation. Photosynth Res. 2014;119(1-2):101-117. doi
10.1007/s11120-013-9874-6

Yan J., Liu J., Yang S., Jiang C., Liu Y., Zhang N., Sun X., … Liu Y.,
Sun Z., Qi M., Wang F., Li T. Light quality regulates plant biomass
and fruit quality through a photoreceptor-dependent HY5-LHC/
CYCB module in tomato. Hortic Res. 2023;10(12):uhad219. doi
10.1093/hr/uhad219

Zhang Q., Ma C., Wang X., Ma Q., Fan S., Zhang C. Genome-wide
identification of the light-harvesting chlorophyll a/b binding (Lhc)
family in Gossypium hirsutum reveals the influence of GhLhcb2.3
on chlorophyll a synthesis. Plant Biol (Stuttg). 2021;23(5):831-842.
doi 10.1111/plb.13294

Zhao S., Gao H., Luo J., Wang H., Dong Q., Wang Y., Yang K., Mao K.,
Ma F. Genome-wide analysis of the light-harvesting chlorophyll
a/b-binding gene family in apple (Malus domestica) and functional
characterization of MdLhcb4.3, which confers tolerance to drought
and osmotic stress. Plant Physiol Biochem. 2020;154:517-529. doi
10.1016/j.plaphy.2020.06.022

